# A Cautionary Note for COVID-19 Survivors: Potential Long-term Risk for Alzheimer’s Disease

**DOI:** 10.34297/ajbsr.2020.09.001415

**Published:** 2020-07-15

**Authors:** Yuxiang Sun

**Affiliations:** Department of Nutrition, Texas A&M University, USA

## Opinion

The COVID-19 pandemic has caused great loss of human lives, massive burdens on health care systems, and enormous damage to economies worldwide. COVID-19, caused by coronavirus 2 (SARS-CoV-2), not only severely damages the respiratory system, but also affects a wide range of organs such as pancreas, kidneys, liver and brain [1]. Since we are still in the midst of the pandemic, the current focus has been primarily on treating the life-threatening symptoms and reducing mortality. However, it is also important to note that emerging evidence shows that many COVID-19 survivors have lingering symptoms of recurrent cough, chronic fatigue, depression, memory loss, etc. for months after the initial infection. This is a very troubling phenomenon, but so far it has gotten little attention. It is clear that SARS-CoV-2 can infect people all ages. While it causes higher mortality in the elderly and people with underlying health issues, recent data in the US and Europe show that an increasing number of young people become ill from the virus, many are healthy individuals in their 20s and 30s. Some young patients are so sick to require hospitalization; some even need ventilators and life support. This new development indicates that youth is not invulnerable to SARS-CoV-2 and surviving COVID-19 may not mean a clean bill of health. It is a harmful to assume that COVID-19 in young people will be short-lasting and mild. Since SARS-CoV-2 is a virus only identified within the past few months, its long-term health consequences are unknown. However, there are ample data showing that other viruses, such as HIV, herpes, chickenpox etc., remain present in the body for years, and the carriers may experience outbreaks when their immune system is weak and are vulnerable to many illnesses. At this stage, as the COVID-19 pandemic unfolds, it is important to keep in mind how little we truly know about this vastly complicated disease. Given the long life expectancy of young individuals and the huge number of infected young people, it is strategically vital to investigate the long-term sequalae of COVID-19 in order to proactively prevent/mitigate human suffering and medical costs in the future. Alzheimer’s Disease (AD) is the most common and devastating neurodegenerative disease, severely affecting quality of life and posing paramount socioeconomic challenges [2]. This opinion article will primarily discuss the potential long-term adverse neuropsychological complications of COVID-19, specifically focusing on the risk of dementia and AD. As we do not have long-term data on COVID 19, the discussion is extrapolated based on scientific knowledge of other viral infections and our current understanding of AD pathogenesis and pathophysiology.

SARS-CoV-2 is a novel virus, humans have no specific immunity to it, so the outcome of the disease is primarily reliant on the generic immune fitness of individuals. Our bodies rely on two different types of immunity to defend against pathogens: adaptive and innate immunity. Adaptive immunity, involving highly specific B-cells and T-cells, is a better-known responsive mechanism reacting to a particular pathogen associated with antibody production. Innate immunity, involving immune cells such as macrophages and microglia, is the first line of defense of the body and a non-specific defense mechanism. Macrophages in peripheral tissues and microglia in brain are the first responders to insults/injury, and are the key drivers of inflammation. Macrophages and microglia have a unique property of plasticity: they undergo dynamical reprogramming in response to environmental cues, polarizing to either pro-inflammatory or anti-inflammatory subtypes [3,4]. In response to an infection/injury, immune cells trigger immune responses and inflammation, which are followed by 3 possible outcomes:

1. The immune system successfully resolves the acute inflammation, the tissue damage is stopped and tissue repair takes place.
2. The inflammation is further escalated and, the massive inflammatory response indistinguishably attacks all tissues, causing collateral damage.
3. The inflammation is attenuated but not completely resolved, converting to chronic inflammation, which may lead to long-term complications and increased susceptibility to various diseases.

In response to virus-induced attack, macrophages and microglia shift to a pro-inflammatory state to produce pro-inflammatory cytokines; the prolonged presence of pro-inflammatory cytokines, sometimes manifesting into a “cytokine storm, causes serious tissue damage. Immunopathogenesis of SARS-CoV-2 infection elicits virus-induced tissue destruction, innate immune response, surge of pro-inflammatory cytokines, and priming of adaptive T-and B-cell immune responses [5].

Sepsis is a life-threatening condition resulting from the invasion of harmful microorganisms such as coronavirus in the blood or tissues. Septic shock is the most severe stage of sepsis when immune system robustly responds to the infection to lead to massive organ failure. Developing data show that severe COVID-19 cases commonly show compromised immunity and uncontrolled inflammation, which can develop into septicemia, sepsis, and septic shock [6–9]. Among hospitalized COVID patients, 59%-70% developed sepsis; of non-survivals, 100% had sepsis. Septic shock is the major cause of death for COVID patents [6–9]. It has been reported that COVID-19 patients show a wide range of neurological manifestations in the central and peripheral nervous systems due to direct damage by the virus, as well as indirect damage caused by cytokine storm, impaired blood-brain barrier and/or blood clots-induced strokes [10]. Many COVID patients show “NeuroCovid” symptoms such as post-sepsis syndrome, post-traumatic stress disorder, anxiety, depression, insomnia, cognitive decline, delirium, mania, etc. long after hospital discharge [1,10]. It is known that people who survived sepsis have weakened immune systems in the weeks and months following the initial bout of sepsis, making them more vulnerable to various infections and diseases [11]. A study of SARS-Cov1 showed a significant increase of a wide spectrum of neuropsychiatric sequelae 31-50 months after the acute infection: post-traumatic stress disorder (39%), depression (36.4%), obsessive-compulsive disorder (15.6%), and panic disorders (15.6%) [12]. Based on the known pathophysiology of COVID-19 and sepsis, it is reasonable to anticipate that there likely will be long-term neuropsychological complications in COVID-19 patients who had sepsis.

Dementia, a brain disorder of progressive cognitive impairment, increases with age and is a hallmark of AD [13–15]. Recent clinical findings suggest that there is significant correlation between past septicemia history and dementia in later life [14]. Intriguingly, it was found that the younger septicemia survivors show a higher risk of dementia than older septicemia survivors after subtracting the expected age-associated cases [14]. New findings indicate that neuroinflammation, primarily governed by microglia, has major roles in pathogenesis and prognosis of dementia and AD [16,17]. In response to insult/injury, microglia shift from a resting state to an activated state and produce pro-inflammatory cytokines; microglia ingest and degrade dead cells and secrete pro-inflammatory cytokines to destroy neuronal synapses, which leads to memory loss [18–20]. It is commonly accepted that exposure of hazardous factors in early life can have profound consequences for a person’s long-term health [21]. New evidence suggests that innate immunity is much more specialized and has persistent and lasting effects than previously recognized [3,22–24]. Other coronaviruses have been shown to contribute to pathogenesis of demyelination and neurodegeneration [5]. It is possible that SARS-CoV-2 may increase neuroinflammation by direct viral injury and microglial reprogramming, which then reshape brain immunity and lead to neural dysfunctions. Based on nascent reports of cognitive symptoms in COVID-19 patients and the known complications of other coronaviruses, it is logical to predict that some COVID-19 survivors may have cognitive impairment and increased incidences of dementia and AD later in life.

Early detection is extremely important for prevention and treatment. To prevent future public health catastrophe, it is critical do longitudinal follow-ups of COVID-19 patients and require the inclusion of COVID-19 infection history as part of the patients’ medical records. For COVID patients who have recovered from septicemia, it would be prudent to pay attention to diet, exercise, and psychological well-being in order to boost their immune resilience. Key inflammation markers such as C-reactive Protein (CRP) and interleukin 6 (IL-6) are known as reliable biomarkers of inflammation [10]. Physicians should order blood tests such as CRP and IL-6 to monitor systemic inflammation, and do COVID-19 PCR and/or serology tests to assess the re-occurrence of the disease. Patients experiencing cognitive issues such as poor memory, attention deficit, or slowness in processing information, 6-8 months after their hospital discharge, should see a neurologist. Neurologists may conduct behavioral assessments to evaluate memory/learning and depression/anxiety, as well as neuroimaging to determine neuropsychological dysfunctions and AD risk.

To conquer the COVID-19 pandemic, we must have a full understanding of the breadth and depth of this complex virus. Over the past several months of the pandemic, “flattening the curve” of the infection spike has become a very familiar phrase. There is likely also to be a long-term disability curve of COVID-19 throughout the lifespans of the survivors. Only when we have flattened the curves of both short- and long- terms, we can then truly say we have defeated COVID-19.
